# Announcement: IUPAC 8th International Symposium on Macromolecule-Metal Complexes (MMC-8)

**DOI:** 10.1155/MBD.1998.317

**Published:** 1998


					IUPAC
8TH INTERNATIONAL SYMPOSIUM ON
MACROMOLECULE-METAL COMPLEXES
(MaC-S)
M 8
Organized by the Division Macromolecular Complexes of Waseda University under the Auspices of
The Chemical Society of Japan and The Society of Polymer Science, Japan
September 5-8, 1999
Ibuka-Memorial Hall, International Conference Center,
Waseda University, Tokyo
Invitation
The organizing committee invites all interested scientists to participate to the 8th International
Symposium on Macromolecule-Metal Complexes. The symposium will be held at the International
Conference Center of Waseda University on September 5-8, 1999.
Scope of the Symposium
This symposium is intended to provide a forum for the expositions and discussions about the most
recent developments on macromolecule-metal complexes (MMC).
During the last 20 years, this research field has spread significantly especially to the role of polymers
on the electronic properties of the central metal moieties. From the interest in the relationship
between this effect and technologies, the international symposia on MMC have been held since
1985, biyearly.
Scientific Program
This symposium will include several basic and applied topics in the field of advanced MMC:
1. Preparation, characterization and fundamental aspects.
2. Macromolecules for advanced technologies.
a) electron-, ion-conductors; b) separation, adsorption, transport of gas molecules;
c) electronic-, magnetic-, photonic-properties; d) catalysis and photocatalysis; e)liquid crystals; f)
biological-, medical- and environmental-use.
Chairman
E. Tsuchida (Waseda University, Tokyo)
International Advisory Board
R. Barbucci (Siena)
E. A. Bekturov (Almaty)
J. Castells (Barcelona)
F. Ciardelli (Pisa)
B. M. Culbertson (Ohio)
A. A. Efendiev (Sumgait)
A. J. Epstein (Columbus)
J. H. Fuhrhop (Berlin)
D. Hill (Brisbane)
Organizing Committee
H. Inoue (Yokohama)
Y. Kurusu (Tokyo)
K. Matsumoto (Tokyo)
H. Nishide (Tokyo)
H. Hirai (Tokyo)
G. H. Hsiue (Taipei)
Y. Y. Jiang (Beijing)
M. Kaneko (Mito)
E. A. Karahanov (Moscow)
Y. Kurimura (Mito)
M. Lewin (New York)
Y. Okamoto (New York)
J. Reedijk (Leiden)
H. Nishihara (Tokyo)
H. Ohno (Tokyo)
I. Okura (Yokohama)
N. Oyama (Tokyo)
G. L. Rempel (Waterloo)
D. C. Sherrington
(Glasgow)
H. Shirai (Ueda)
I. Shopov (Sofia)
N. Toshima (Onoda)
E. Tsuchida (Tokyo)
D. WShrle (Bremen)
K. Ueyama (Toyonaka)
S. Takahashi (Suita)
S. Tokita (Urawa)
N. Toshima (Onoda)
317
Program Committee
K. Hanabusa (Ueda)
M. Kaneko (Mito)
T. Osaka (Yokohama)
Secretariat
T. Komatsu K. Miyatake
Language
K. Osakada (Yokohama)
K. Segawa (Tokyo)
K. Yamamoto (Yokohama)
H. Nishide
S. Yamashita (Tokyo)
M. Watanabe (Yokohama)
The official language of the Symposium is English.
Registration
US$ 300.- for each active participant including the book of abstracts. US$150- for accompanying
participants (including graduate student). The deadline is the end of April 1999 for the abstracts, and
the end of May 1999 for the final registration. Those planning to attend the symposium are kindly
requested to contact the organisers.
Participants requiring a visa to Japan should inquire at their local Japanese Consulate.
Second Circular
Details on the program and instructions for abstract will be provided in the second circular, which will
be mailed by the end of December 1998.
Correspondence
Symposium Secretariat, MMC-8
Bldg.55S Rm.701, ARISE, Waseda University
Tokyo 169-8555, Japan
Phone: +81 3-5286-3120 / Fax: +81 3-3205-4740
e-mail" macro@ mn.waseda.ac.jp
318

				

